# Cloning and expression of the NS1 protein of dengue virus in a prokaryotic system

**DOI:** 10.1186/1753-6561-8-S4-P24

**Published:** 2014-10-01

**Authors:** Danielle Ferreira de Oliveira, Lívia Érika Carlos Marques, Bruno Bezerra da Silva, Sergio Rodriguez Málaga, Lia Magalhães de Almeida, Tatiane Rodrigues Oliveira, Maria Izabel Florindo Guedes

**Affiliations:** 1 Universidade Estadual do Ceará, Fortaleza, Brazil; 2 Universidade Federal do Pará, Belém, Brazil

## Background

Dengue virus nonstructural protein 1 (NS1) is a highly conserved glycoprotein involved in the production of infectious virus and the pathogenesis of dengue disease [1]. Serum or plasma DENV NS1 level has been found to correlate with viremia titer and disease severity. It can be found in the peripheral blood circulation for up to 9 days from illness onset, but can persist for up to 18 days from illness onset in some cases. Thus NS1 detection offers a larger window of opportunity for diagnosis of dengue compared with virus isolation, RT-PCR or NASBA [2]. In this context, the aim of this work has been the establishment of conditions for expression and purification of the recombinant protein NS1 of dengue virus serotype 2 produced in *E. coli *for further development of a serological diagnostic method at low cost.

## Methods

The gene encoding the NS1 protein was amplified through RT-PCR and subcloned into the expression vector pET-28a for expression of the recombinant protein. The corresponding recombinant plasmid was transformed into the NS1 protein in bacteria *E. coli *strain BL21 (DE3) and the clones obtained were expanded and induced with different concentrations of IPTG at 37°C for analysis of the expression of recombinant proteins. After lysis of the bacterial culture, the protein fractions collected (supernatants and precipitates) were analyzed by SDS-PAGE and immunoblotting with monoclonal antibodies (anti-His6).

## Results and conclusions

The SDS-PAGE and immunoblotting analyses revealed the presence of proteins of approximately 45 kDa in the precipitates. This result indicated the formation of inclusion bodies, which is commonly found for proteins produced in prokaryotic system. Thus, one of the perspectives of this work is to standardize methods for the obtainment of soluble proteins under suitable conditions for immunological studies.
